# Efficient Decolorization of the Poly-Azo Dye Sirius Grey by *Coriolopsis gallica* Laccase-Mediator System: Process Optimization and Toxicity Assessment

**DOI:** 10.3390/molecules29020477

**Published:** 2024-01-18

**Authors:** Héla Zouari-Mechichi, Jihen Benali, Abdulrahman H. Alessa, Bilel Hadrich, Tahar Mechichi

**Affiliations:** 1 Laboratory of Biochemistry and Enzymatic Engineering of Lipases, National Engineering School of Sfax (ENIS), University of Sfax, Sfax BP1173 3038, Tunisia; hela.zouari@isbs.usf.tn (H.Z.-M.); jihen.benali@enis.tn (J.B.); 2 Department of Biology, Faculty of Science, University of Tabuk, Tabuk 47512, Saudi Arabia; alessiabdulrahman@gmail.com; 3 Department of Chemical Engineering, College of Engineering, Imam Mohammad Ibn Saud Islamic University, IMSIU, Riyadh 11432, Saudi Arabia; bmhadrich@imamu.edu.sa

**Keywords:** laccase, Sirius-grey, azo dyes, 1-hydroxybenzotriazole, decolorization

## Abstract

The textile industry produces high volumes of colored effluents that require multiple treatments to remove non-adsorbed dyes, which could be recalcitrant due to their complex chemical structure. Most of the studies have dealt with the biodegradation of mono or diazo dyes but rarely with poly-azo dyes. Therefore, the aim of this paper was to study the biodegradation of a four azo-bond dye (Sirius grey) and to optimize its decolorization conditions. Laccase-containing cell-free supernatant from the culture of a newly isolated fungal strain, *Coriolopsis gallica* strain BS9 was used in the presence of 1-hydroxybenzotriazol (HBT) to optimize the dye decolorization conditions. A Box–Benken design with four factors, namely pH, enzyme concentration, HBT concentration, and dye concentration, was performed to determine optimal conditions for the decolorization of Sirius grey. The optimal conditions were pH 5, 1 U/mL of laccase, 1 mM of HBT, and 50 mg/L of initial dye concentration, ensuring a decolorization yield and rate of 87.56% and 2.95%/min, respectively. The decolorized dye solution showed a decrease in its phytotoxicity (Germination index GI = 80%) compared to the non-treated solution (GI = 29%). This study suggests that the laccase-mediator system could be a promising alternative for dye removal from textile wastewater.

## 1. Introduction

Dyeing clothes is a prehistoric process. This process involved the application of early natural dyes for furs and textiles of vegetable origin, though some dyes were of animal origin. More complex coloring materials were developed over thousands of years. The woad (natural indigo), for example, was obtained from the plant *Indigofera tinctoria*, and the Tyrian purple was extracted from the gland of a purple snail and developed by the Phoenicians, whereas the Alizarine was taken out from madder Campeachi wood [1]. By the end of the 19th and early 20th centuries, the synthetic dye industry was established in many countries, and thousands of dye molecules were synthesized and produced on a large scale [2].

Despite the chemical diversity of dyes, dye molecules share a common chemical structure. In fact, each dye molecule has four components, namely a chromophore group, an auxochrome group, a solubilizing group, and a matrix. The chromophore groups are responsible for the absorption of the light energy and the creation of the dye’s color through the excitation of electrons. The auxochrome groups help with the dye fixation into the support, while the solubilizing ones ensure the solubility of the molecule in water or organic solvents. The remaining parts of the dye molecule form the matrix or the skeleton [1].

Dyes are classified according to several parameters, including color, chemical structure, application, manufacturer, synthesis route, fastness, and date invented. However, based on their chemical structure and the chromophore groups, the following dye families were identified: azo, anthraquinone, nitroso, nitro, indigoid, cyanine, phthalocyanine, and triphenylmethane [3].

Azo dyes are characterized by two aromatic groups linked to each other by an azo bond (-N=N-). They are classified based on the number of azo linkages, mono azo dyes, diazo dyes, etc. The number of azo groups varies from 1 to 4. Other than textile industries, this family of dyes is used in various fields such as pharmaceuticals, cosmetics, food, paint, paperwork, etc. Their success is mostly due to the stability of coloring, the ease of a coupling reaction between the dyes and the support, the high molar extinction coefficient (capacity to absorb light), the flexibility of the coloring structure, and their adaptability to a variety of applications [4,5,6].

Approximately 70% of the dyes used in the textile industry are of the azo type. However, during the coloring process, non-adsorbed dyes are estimated to be between 15 and 20% and are discharged into the wastewater [7,8,9]. Due to their toxicities, industries using this type of dye are currently attempting to minimize their negative impact on the environment. This includes improving their binding to the matrix or their degradation once discharged into industrial wastewater using biological or physicochemical processes.

Many studies have demonstrated that the released sewage contains, other than dyes, toxic molecules like heavy metals. Once released into the environment, the wastewater may affect both human health and ecosystem [10,11,12]. Many health issues, including cancer, chronic diseases, and skin irritation, have been associated with exposure to azo dyes [10]. Furthermore, the death of aquatic organisms and the stunting of plant growth were mentioned as a consequence of the release of untreated textile wastewater [13]. To treat sewage from the textile industry, many attempts have been made, and many physicochemical methods have been developed (e.g., filtration, adsorption, coagulation/flocculation). Those treatments were mostly used at the outset. Nevertheless, their unwanted outcomes, like the formation of secondary mud, the limited efficacy, and the high cost, have prompted industries to look for alternative biological methods that are especially eco-friendly and low-cost and where plant microorganisms and/or their enzymes can be used. Yeast [14], bacteria [15,16], algae [17], and fungi [18] have widely been used for this purpose. Several studies demonstrated the efficiency of white-rot fungi such as *Trametes versicolor* [19], *Trametes trogii* [20], and *Coriolopsis gallica* [7,21], and other fungi like *Aspergillus niger* [22] in the removal of textile dyes using their enzymes or biomasses.

White-rot fungi secrete a number of oxidoreductases that are involved in lignin depolymerization [23,24]. These oxidoreductases encompass heme-containing peroxidases (manganese, lignin, and versatile peroxidases) and copper-dependent polyphenol oxidases named laccases (E.C. 1.10.3.2). Laccases from white-rot fungi exhibit a higher redox potential (0.720–0.790 V) compared to other fungal, bacterial, or plant laccases (0.400–0.700 V). High redox potential laccases do not oxidize lignin directly but through small aromatic compounds (laccase-mediators) that can attack lignin after their oxidation in the active site [25,26]. Although laccases are widely used, their efficiency in removing pollutants is sometimes limited; this has prompted the use of a laccase-mediator system to enhance the laccase activity. Mediators allowed the active center of the enzyme to interact with large molecules of substrates or substrates with high redox potential. Several laccase mediators have been studied, including mediators of natural molecules (e.g., 3-hydroxy-anthranilic acid, syringaldehyde, vanillin, etc.) or synthetic molecules (such as HBT, TEMPO, Violoric acid, etc.) [27].

In this paper, we aim to study the biodegradation of the four azo-bond dye Sirius grey by the laccase mediator system and to optimize its decolorization conditions using a response surface methodology approach.

## 2. Results

### 2.1. Fungi Isolation and Identification

**Figure 1:**
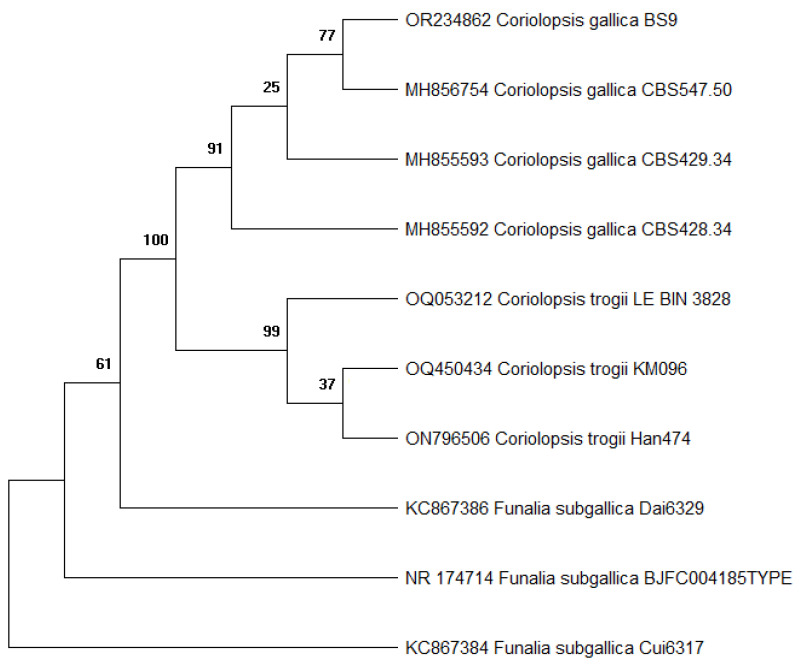
A phylogenetic tree based on ITS rDNA sequences compared to reference sequences from Genbank shows the relationship of strain BS9 to its closest strains.

A pure culture, designated strain BS9, was obtained after the growth of mycelia from fungal crust collected on decayed wood. The internal transcribed spacer (ITS) regions of fungal rDNA have been successfully used for species identification (Figure 1). Blast analysis showed that strain BS9 shares more than 99% similarity of the whole ITS region with *C. gallica* CBS 547.50 and several other *C. gallica* strains represented in GenBank. Based on this and on the morphological characteristics of the collected fungi, strain BS9 was assigned to the species *C. gallica*.

### 2.2. Laccase-like Enzymes Detection and Production

Strain BS9 was tested first for its capacity to produce laccase-like enzymes on a PDA medium in the presence of 5 mM 2,6-dimethxyphenol as a laccase substrate. After fungal growth, the non-colored PDA medium turned orange around the mycelium, indicating the presence of 2,6-DMP oxidizing enzymes. For laccase-like enzyme production, strain BS9 was cultivated in an M7 medium. Enzyme production was induced by 300 µM of CuSO_4_ on the 3rd day of cultivation. Laccase production increased from day 5 (500 U/L) to reach its maximum on day 10 with 3767 U/L. 

### 2.3. Dye Decolorization by Crude Laccase from C. gallica

The capacity of the crude laccase from strain BS9 to decolorize the dye Sirius grey (50 mg/L) was first tested under non-optimized conditions (at 30 °C, pH 5.5, 50 mg/L of dye, and with 1 U/mL of laccase-like activity) in the presence (1 mM) and the absence of laccase mediator (1-hydroxybenzotriazole). Results showed that the laccase could decolorize the dye Sirius grey to a lower extent (48%) without HBT, but when 1 mM of HBT was added to the reaction mixture, the decolorization increased up to 81% in 4 h. 

### 2.4. Optimization of Dye Decolorization Conditions

To increase the efficiency of the process and decrease its cost, the dye decolorization conditions were optimized using a response surface methodology approach. Four essential parameters that are known to influence substrate oxidation by laccase were tested to optimize this process. These parameters were pH, dye concentration, HBT concentration, and enzyme concentration. Two responses were recorded: dye decolorization yield and rate. The obtained experimental results of decolorization yields and rates ranged between 40.14 ± 2.03% (Run 22)—87.02 ± 1.33% (Run 18) and 1.29 ± 0.06%/min (Run 6)—3.63 ± 0.12%/min (Run 23), respectively (Table 6). In fact, among 27 conditions, we have 20 presenting a yield higher than 55% of decolorization. Moreover, the decolorization rates ranging between 1.29 ± 0.06%/min–3.63 ± 0.12%/min are very interesting to study the rapidity of the process, i.e., the kinetics of the decolorization. Those results can reveal the adequacy of the selected levels range for all factors (also called independent variables) at the beginning of this study. In addition, it is clear that all results of decolorization yields and rates are repetitive since their standard deviation is very low (0.04–2.81% and 0.01–0.24%/min, respectively). This proves that the process of the enzymatic decolorization of Sirius grey can be considered stable and without technical problems such as feasibility.

### 2.5. Modeling Dye Decolorization Yield

Equation (1) presents the model coefficients relative to the decolorization yield of Sirius grey (${\hat{y}}_{1}$):

(1) $${ŷ}_{1}\left(\%\right)=79.30+8.84\cdot x_{1}+2.44\cdot x_{2}-5.78\cdot x_{3}+7.14\cdot x_{4}\;–3.52\cdot x_{1}^{2}-19.69\cdot x_{2}^{2}-1.29\cdot x_{3}^{2}\;-4.48\cdot x_{4}^{2}+3.19\cdot x_{1}\cdot x_{2}+2.07\cdot x_{1}\cdot x_{3}\;–1.15\cdot x_{1}\cdot x_{4}-3.31\cdot x_{2}\cdot x_{3}+2.66\cdot x_{2}\cdot x_{4}+2.27\cdot x_{3}\cdot x_{4}$$

where $x_{1}$, $x_{2}$, $x_{3}$, $x_{4}$ are the coded levels (see Table 5) of initial HBT concentration (factor A), pH (factor B), initial dye concentration (factor C), and initial enzyme concentration (factor D), respectively. 

Table 1 presents the ANOVA results of Sirius grey decolorization yield (Equation (1)) and of each factor and interaction. It is clear that the regression using the determined model presents a high degree of significance since *p* < 0.001 (see first row of Table 1). This is confirmed by the high values of R² = 91.63% and R²_Adj_ = 89.86% and by the low value of RMSE = 4.29% (in terms of percentage of decolorization yield).

In addition, the coefficients in Equation (1) indicated a highly significant influence of the selected factors on the Sirius grey decolorization, as confirmed by the importance of their values (in decreasing order—see Equation (1) and Table 1): (1) initial HBT concentration in linear (Coefficient = 8.84; *p* < 0.001) and in quadratic (Coefficient = −3.52; *p* = 0.002 < 0.05) term; (2) initial enzyme concentration in linear (Coefficient = 7.14; *p* < 0.001) and in quadratic (Coefficient = −4.84; *p* < 0.001) term; (3) initial dye concentration only in linear term (Coefficient = −5.78; *p* < 0.001); and pH in linear (Coefficient = 2.44; *p* = 0.001 < 0.05) and in quadratic (Coefficient = −19.69; *p* < 0.001) term. Moreover, three interactions among six present a very significant effect on the studied decolorization yield. All these significant interactions involved the pH of the reaction mixture. Interaction were as follows: initial HBT concentration × pH (*p* = 0.012 < 0.05); pH × initial dye concentration (*p* = 0.009 < 0.05); pH × initial enzyme concentration (*p* = 0.036 < 0.05). 

**Figure 2:**
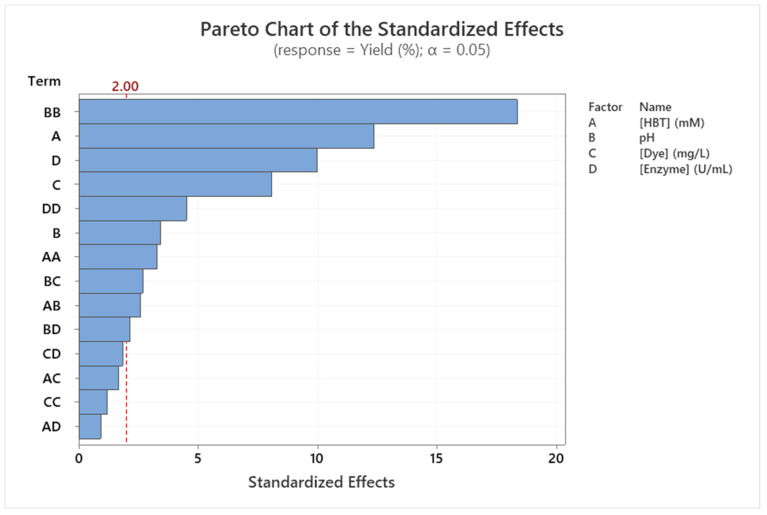
Pareto chart of the standardized effects of the Sirius grey decolorization yield (%) (*p* < 0.05).

The statistical analyses presented in Table 5 were confirmed graphically using a Pareto chart (Figure 2).

**Figure 3:**
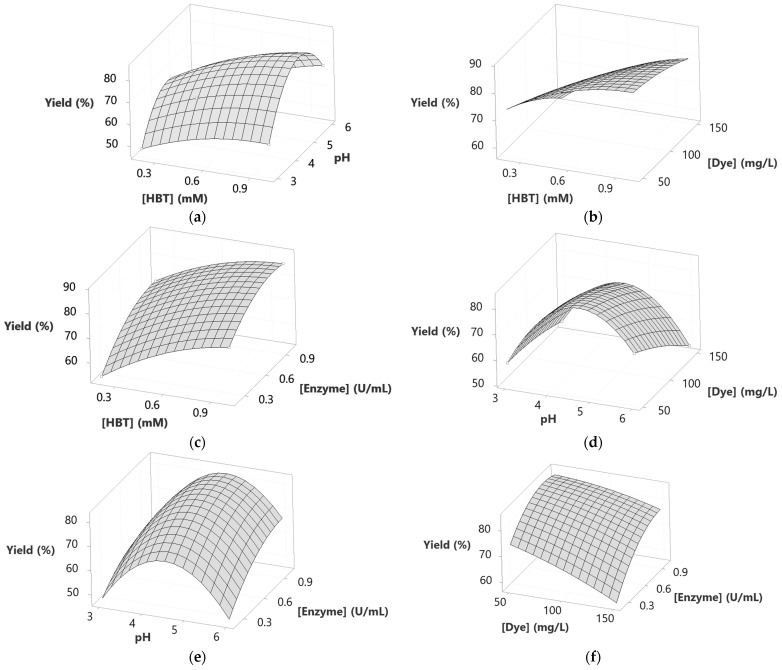
3D-Surfaces response of Sirius grey decolorization yield as a function of (**a**) [HBT] and pH; (**b**) [HBT] and [Dye]; (**c**) [HBT] and [Enzyme]; (**d**) pH and [Dye]; (**e**) pH and [Enzyme] (**f**) [Dye] and [Enzyme]. All other factors are fixed at the central level.

3D response surface diagrams (Figure 3) show that operating at relatively high HBT concentrations, regardless of the other influencing factors, increased the decolorization yield. The interaction between HBT and pH (Figure 3a) shows that the maximum decolorization yield (80%) was reached for 1 mM of HBT and for a pH range between 4 and 5. In addition, increasing the HBT from 0.2 to 1 mM enhanced the yield of decolorization regardless of the dye concentration (Figure 3b). This was also the case for the HBT concentration and enzyme concentration, where the responses became higher when both HBT concentration and enzyme concentration were increased from 0.2 to 1 mM and from 0.2 to 1 U/mL, respectively (Figure 3c).

The effect of pH combined with dye concentration is shown in Figure 3d. The maximum decolorization yield was obtained for a pH range between 4 and 5 and for the lowest dye concentration value. The same parabolic behavior was obtained while combining the effect of pH and enzyme concentration, where 80% of the decolorization yield was reached for the same range of pH and for the highest enzyme concentration (1 U/mL) (Figure 3e). Increasing the enzyme concentration from 0.2 to 1 U/mL while minimizing the concentration of dye from 150 to 50 mg/L had a positive effect on the response, as shown in Figure 3f. All the above behaviors are already described with the corresponding coefficients in the determined model (see Equation (1)).

### 2.6. Modeling Dye Decolorization Rate 

Equation (2) presents the model coefficients relative to the Sirius grey decolorization rate (${\hat{y}}_{2}$):

(2) $$\begin{array}{c} {ŷ}_{2}\left(\%/\min\right)=2.07+0.11\cdot x_{1}-0.47\cdot x_{2}-0.13\cdot x_{3}+0.59\cdot x_{4}+0.04\cdot x_{1}^{2}+0.37\cdot x_{2}^{2}\\ \;+0.03\cdot x_{3}^{2}+0.12\cdot x_{4}^{2}+0.08\cdot x_{1}\cdot x_{2}+0.06\cdot x_{1}\cdot x_{3}+0.02\cdot x_{1}\cdot x_{4}\\ \;-0.06\cdot x_{2}\cdot x_{3}+0.14\;\cdot x_{2}\cdot x_{4}+0.07\cdot x_{3}\cdot x_{4} \end{array}$$

where $x_{1}$, $x_{2}$, $x_{3}$, $x_{4}$ are the coded levels of initial HBT concentration, pH, initial dye concentration, and initial enzyme concentration. 

ANOVA results corresponding to the integrality-determined model of Sirius grey decolorization rate (Equation (2)) and to each factor, as well as factor interactions, are presented in Table 2. The value of *p* < 0.001 confirms that the corresponding regression has an interesting degree of significance. The high values of R² = 92.48% and R²_Adj_ = 90.88%, and the low value of RMSE = 0.173%/min confirm the regression/model quality.

**Figure 4:**
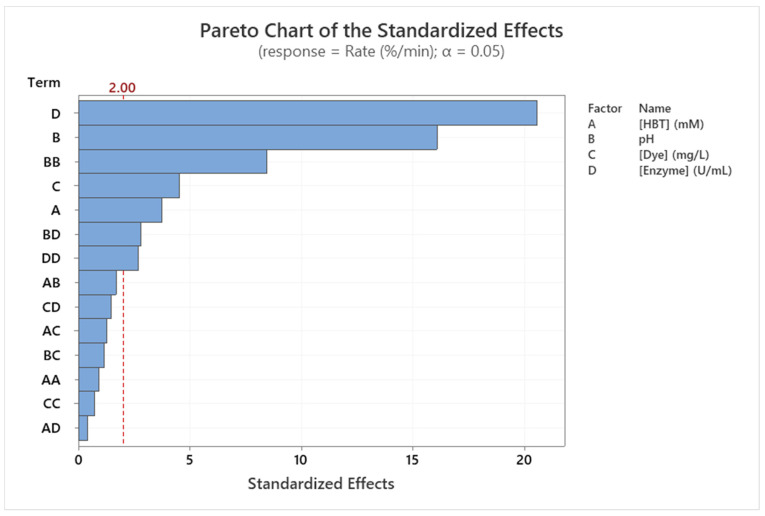
Pareto chart of the standardized effects of the Sirius grey decolorization rate(%/min) (*p* < 0.05).

ANOVA test analysis (Table 2) and Pareto chart (Figure 4) already show the significant influence of the tested factors on the decolorization rate (in linear and quadratic terms). In fact, all factors influence linearly the decolorization rate of Sirius grey (*p* < 0.001). Only the quadratic influence of pH (Coeff. = 0.37; *p* < 0.001) and enzyme concentration (Coeff. = 0.12; *p* < 0.001) are considered significant on the Sirius grey decolorization rate. Moreover, only the interaction between pH and enzyme concentration is significant (*p* < 0.001). This was confirmed by Pareto Chart analysis (Figure 4).

**Figure 5:**
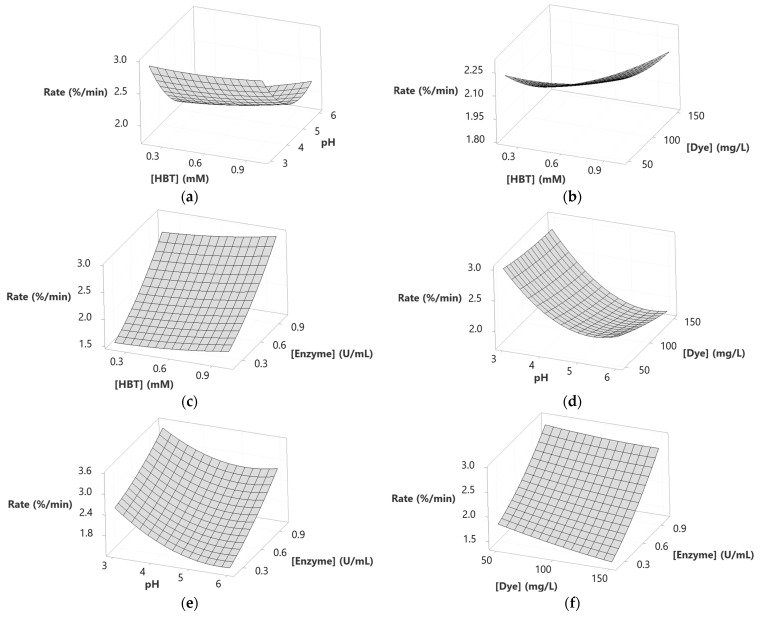
3D-Surfaces response of Sirius grey decolorization rate as a function of (**a**) [HBT] and pH; (**b**) [HBT] and [Dye]; (**c**) [HBT] and [Enzyme]; (**d**) pH and [Dye]; (**e**) pH and [Enzyme]; (**f**) [Dye] and [Enzyme]. All other factors are fixed at the central level.

Figure 5 presents the 3D-surface response of Sirius grey decolorization rate. Those graphics translate the results of the model describing the dependence of the decolorization rate (as a response) to the studied variables. It seems that the suitable pH to maximize the decolorization rate was 3 (Figure 5a,d,e). In fact, Figure 5a showed that processing the decolorization at pH 3 and at any level of HBT concentration yielded a constant and higher decolorization rate. Moreover, the same behavior was observed at pH 3 and at any dye concentration. It decreased as the pH increased from 4 to 6, as shown in Figure 5d. Decreasing the pH from 6 to 3 while increasing the enzyme concentration improved the decolorization rate to reach 3.5%/min for 1 U/mL of enzyme and pH 3 (Figure 5e). However, increasing the HBT concentration while maintaining the pH and enzyme concentration constant (at 4.5 and 0.6 U/mL, respectively), regardless of the dye concentration, improved the decolorization rate (Figure 5b). According to Figure 5c, the decolorization rate reached its maximum (2.9%/min) when the HBT concentration increased from 0.2 to 1 mM, and the enzyme concentration increased from 0.2 U/mL to 1 U/mL. The interaction between dye concentration and enzyme concentration presented in Figure 5f shows that maximizing the enzyme concentration to 1 U/mL improved the rate of decolorization at different dye concentrations (50, 100, 150 mg/L). The impact of dye concentration was noticeable when processing at a low enzyme concentration. However, this distinction became less significant at higher concentrations.

### 2.7. Optimization of Responses 

**Figure 6:**
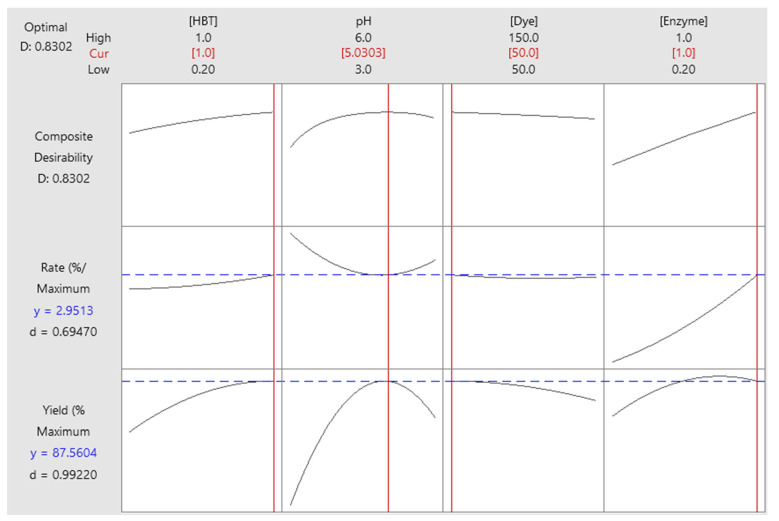
Optimal conditions determination for Sirius grey decolorization.

Figure 6 represents the results of the optimization of responses. In fact, better decolorization is defined by the maximum values of both responses: decolorization yield and decolorization rate. Indeed, a high Sirius grey decolorization could be obtained by fixing the initial HBT concentration to a maximum value (1 mM), pH to 5, initial dye concentration to a minimum value (50 mg/L), and initial enzyme concentration to a maximum (1 U/mL). In this case, the decolorization yield reached 87.56% and the decolorization rate 2.95%/min. In order to experimentally confirm the optimization results, the decolorization reaction was performed under optimal conditions in triplicates. The obtained results were 83.50 ± 0.50% and 3.69 ± 0.11%/min for decolorization yield and rate, respectively. 

### 2.8. Evaluation of the Toxicity of Treated and Untreated Dye

**Figure 7:**
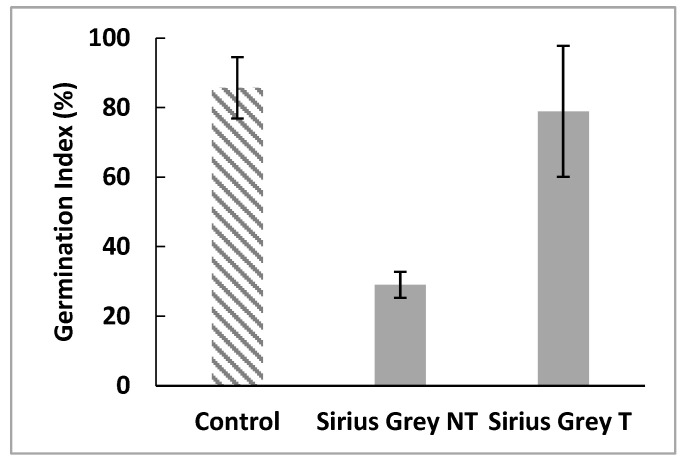
Effects of treated and non-treated Sirius grey solutions on the germination index of *Raphanus sativus* (T: treated; NT: non-treated).

Phytotoxicity tests were performed using radish seeds, which were incubated in treated and untreated dye solutions. Germination indexes were calculated in order to determine the inhibition caused by the dye or its degradation products. Distilled water was used as a control. Figure 7 shows that the treatment of the dye by a cell-free supernatant of *C. gallica* helps to decrease the phytotoxicity of Sirius grey by increasing GI from 29 ± 3.75% to 78.95 ± 18.84%. These results indicate that although Sirius grey is phototoxic, its degradation does not yield toxic metabolites. 

## 3. Discussion

Strain BS9 shares more than 99% similarity of its ITS1-ITS4 region of rDNA with members of the species *C. gallica*. This fungus is known for its capacity to grow on several woods to produce ligninolytic enzymes [7,28]. Based on this fact, *C. gallica* was also shown to degrade many pollutants, including dyes [7,21], hydrocarbons [29], phenols, and bisphenol A [30]. Recently, *C. gallica* was shown to be able to degrade antibiotics [31]. In the same work, proteomic analysis showed the presence of one major secreted laccase, although there were several laccase genes in the genome.

Decolorization of a wide range of synthetic and textile dyes using laccases from basidiomycetes has been investigated in recent years. For this reason, we used *C. gallica* for the decolorization of Sirius grey; the latter belongs to the azo compounds that contain one or more azo groups (N=N), and most of them are xenobiotics [32]. By using the culture filtrate from *C. gallica*, 48% decolorization of Sirius grey was achieved. This decolorization yield was improved by adding 1 mM of HBT to the reaction mixture. Under these conditions, the decolorization yield increased to 81%. Therefore, the use of a mediator (such as HBT) is necessary, especially for certain laccases with low redox potential or in case the substrate is highly recalcitrant. Indeed, laccase mediators are low molecular weight molecules with a significant redox potential, enabling them to act as an electron messenger between the substrate and the enzyme [27].

The experimental design that was performed used four variable factors, namely: initial enzyme concentration, initial dye concentration, initial HBT concentration, and pH. Ben Ayed et al. [7] found that these factors had a significant effect on Reactive black 5 (RB5) decolorization using a laccase-like activity of cell-free supernatant from *C. gallica*. The optimized conditions obtained for laccase concentration, HBT concentration, and pH were 1 U/mL, 50 mg/L, 1 mM, and pH 5, respectively, with a maximum decolorization yield of 87%.

In this study, the crude laccase of *C. gallica* was used in the decolorization experiments, and the presence of four azo groups in the Sirius grey makes its treatment more challenging. The decolorization rate obtained here (87% after 4 h) is significant when compared to the results reported by Daâssi et al. [21]. In their study, they used partially purified *C. gallica* laccase for the treatment of three different groups of dyes. However, the RB5 and Bismarck brown R (BBR), which are diazoic dyes, did not show significant decolorization rate. Concerning BBR, the rate was approximately 47.1% over 24 h, whereas, for RB5, this rate did not exceed 70%, even after 24 h of incubation in the presence of 1 mM HBT.

To identify the influence of the interactions between studied factors on decolorization yield and rate, 3D-surface responses were designed (Figure 3). Increasing the HBT concentration to its highest level (1 mM), followed by the increase of pH to 4–5, resulted in enhancing decolorization yield to a level of 80% (Figure 3a). This effect was observed in the interactions between pH × dye concentration and pH × enzyme concentration (Figure 3d,e, respectively). However, increasing the pH beyond 5 led to a reduction of decolorization yield, and this aligns with Forootanfar et al.’s [33] observations. These findings were explained by the fact that hydroxide anions could bind to the enzyme at acid pH, and this negatively affects the electron transfer. In contrast, Aksu and Tezer [34] considered the possibility of basic azo dyes becoming charged positively at higher pH, and this affects their interactions with the mediator and enzyme. The effect of this interaction on the decolorization rate showed that the highest rate was achieved at pH 3, independent of the concentration of the other factors (Figure 5a,d,e). This can be explained by the fact that pH 3 matches the optimum pH for the used enzyme, allowing the decolorization to reach a high speed. This aligns with previous studies claiming that fungal laccases are active at the 3–5 pH range [7,35]. In addition, the stability of the dye can be affected at pH 3, increasing the rate of decolorization even more, as reported before by Yin et al. [36] and Ben Ayed et al. [7].

Figure 3b shows the interaction between HBT concentration × dye concentration at pH 4.5 and 0.6 U/mL of enzyme. Increasing the HBT concentration to its maximum level at different dye concentrations resulted in an increase in the decolorization rate from values less than 1.80%/min (for 0.2 mM of HBT and 150 mg/L of dye) to values more than 2.25% (for 1 mM of HBT and 50, 100 and 150 mg/L of dye). This means that higher HBT concentrations improved the dye oxidation by laccase. However, at 150 mg/L dye concentration (and 1 mM of HBT), the decolorization yield decreased slightly, and this was likely due to enzyme and HBT saturation. As a matter of fact, excessive dye concentration can lead to enzyme inhibition and/or unproductive reactions by intensifying competition for the enzyme’s active sites and substrate saturation. This hypothesis about enzyme-substrate concentration was discussed by Cifçi et al. [37]. The effect of saturation can also be seen in the enzyme concentration × HBT concentration interaction (Figure 3c). In fact, increasing HBT concentration up to 0.8 mM and that of the enzyme to 0.8 U/mL could lead to an increase in the decolorization yield reaching 85%, but a higher concentration of enzyme and HBT led to a slight decrease attaining 80%. Accordingly, the presence of the enzyme and the mediator facilitates an efficient dye cleavage.

The effect of the interaction of dye concentration × enzyme concentration on the decolorization yield is depicted in Figure 3f. It can be seen that treating high dye concentrations at low enzyme levels (and at an HBT concentration of 0.6 mM) might cause a saturation effect of the enzyme and, therefore, of the decolorization yield. However, when the substrate concentration was decreased while increasing that of the enzyme, the decolorization yield was enhanced. This suggests that higher enzyme concentrations provide more active sites for the degradation of the dye. 

With regard to the decolorization rate, Figure 5 represents the effects of factor interactions. Figure 5b illustrates the interaction between HBT and dye concentrations. The lowest decolorization rate (1.8%/min) was observed for a minimal HBT concentration (0.2 mM) and a maximum concentration of Sirius grey concentration (150 mg/L). Conversely, a high response (2.2%/min) was obtained with a maximum mediator level (1 mM of HBT) and at different dye concentrations or at low concentrations of both dye and HBT. These results were expected since they align with the mediator’s role in improving electron transfer between the enzyme and substrate and enhancing the treatment rate [27,38]. In fact, higher dye concentrations necessitate greater mediator concentrations to accelerate the decolorization rate. The need for a mediator was also shown in the interaction between HBT concentration × enzyme concentration (Figure 5c). Indeed, this interaction showed that a higher response of approximately 3%/min was reached at higher concentrations of both factors (1 mM of HBT and 1 U/mL of enzyme). So, as the levels of the enzyme and mediator were increased, the rate of dye decolorization was accelerated. The dye concentration × enzyme concentration interaction presented in Figure 5f exhibited linearity, showcasing a high response (2.8%/min) at 1 U/mL of laccase for various dye concentrations, maintaining a pH of 4.5 and 0.6 mM of HBT. Increasing enzyme concentration means increasing the active site number, which boosts the decolorization rate [39].

As textile industry effluents could be used for the irrigation of some crops [40,41], it is necessary to evaluate their phytotoxicity. In the present study, the toxicity of the treated and untreated Sirius grey solution was evaluated by measuring the germination index of radish seeds. It was found that the Germination Index (%IG) was significantly increased after the treatment of Sirius grey by the supernatant of *C. gallica* compared to the dye solution. This indicates that the treatment with laccase has effectively minimized the toxicity of the dye to lower levels compared to that of untreated dye.

## 4. Materials and Methods

### 4.1. Chemicals

**Figure 8:**
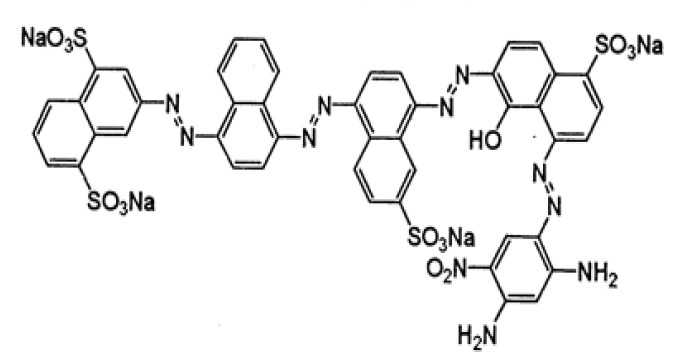
The chemical structure of Sirius grey GB (Direct black 76) is the four azo bonds and four sulfonate groups dye.

2,6-Dimethoxyphenol (DMP) and 1-hydroxybenzotrizole (HBT) were obtained from Sigma-Aldrich, Saint Quentin Fallavier, France. Sirius grey GB (Bayer Global, Leverkusen, Germany) was obtained from a textile factory located in Ksar Helal (Tunisia). Their properties are summarized in Table 3. Sirius grey chemical structure is depicted in Figure 8.

### 4.2. Media and Culture Conditions

Potato-Dextrose-Agar (PDA) medium was used for a short-term conservation of the fungal strain. After growth at 30 °C, plates were stored at 4 °C and sub-cultured monthly. Laccase production by *C. gallica* was performed in a liquid medium, as described by Ben Ayed et al. [7]. Media were inoculated by glass-beads homogenized mycelium (1%), and laccase production was induced by CuSO_4_ at a final concentration of 300 μM. Cultures were incubated at 30 °C and 160 rpm, and when maximum laccase production was reached (8–10 days), laccase-rich supernatant was separated from biomass by filtration on 3 M filter paper and stored at −20 °C until utilization.

### 4.3. Fungal Strain Isolation and Identification

#### 4.3.1. Isolation 

The fungus used in this study was the newly isolated strain BS9. To isolate this strain, a piece of fungal crust growing on a decayed Eucalyptus (*Eucalyptus globulus* Labill.) wood was inoculated on a PDA medium and incubated at 28 °C. The decaying wood pieces were collected in the region of Bousalem (coordinates 36.653176 N, 8.904304 E), Northwest of Tunisia, during the winter of 2021. The growing mycelium was transferred several times on the same solid medium until a pure culture was obtained. The culture was tested for laccase activity production on a PDA medium supplemented with 0.01% guaiacol. When oxidized, the non-colored substrate turns orange, indicating the production of a phenol oxidase activity.

#### 4.3.2. Fungal DNA Extraction, Amplification and Sequencing

Molecular identification was performed based on the internal transcribed spacer (ITS). Fungal genomic DNA, ITS region amplification, and sequencing were performed as described by Ostrowski et al. [42]. The sequences obtained were deposited in the GenBank database under the accession number OR234862.

#### 4.3.3. Phylogenetic Analysis

CAP3 algorithm (UGENE v.37.0) was used to assemble the obtained reads [43]. For preliminary taxonomic placement, the BLASTn algorithm was used to compare consensus sequences with those in the NCBI nucleotide database. An additional nine reference sequences were retrieved from GenBank to prepare a phylogenetic tree (Table 4). All sequences were aligned using the muscle algorithm as implemented in the SeaView program [44]. Subsequently, the alignments were trimmed in the trimAl program using the automated algorithm [45]. The phylogenetic tree was calculated using the maximum likelihood approach, as described by Koslov et al. [46]. The tree robustness was assessed by bootstrap analyses with 1000 replicates. The isolate was assigned to species based on its position on the phylogenetic tree.

### 4.4. Enzyme Assay

Laccase assay was performed with 10 mM 2,6-dimethoxyphenol (DMP) as substrate in 100 mM tartrate buffer, pH 5 (ε_469 nm_ = 27,500 M^−1^cm^−1^) [47]. One unit of laccase activity was defined as the amount of enzyme oxidizing 1 μmol of substrate per minute. 

### 4.5. Dye Decolorization Experiments 

All experiments were performed in 2 mL disposable cuvettes containing 1.5 mL final reaction volume. The reaction mixture contained 100 mM tartrate buffer pH 3 to 6, HBT, 50 mg/L of dye, and culture filtrate (0.5 U/mL laccase). The reaction was initiated by the addition of culture filtrate. The decolorization was followed by measuring the absorbance of the dye solution at the maximum wavelength (610 nm), as indicated in Table 3. All experiments were performed in triplicates; controls did not contain laccase. The incubation was performed for all experiments at 30 °C for 4 h. The pH of the tartrate buffer, dye concentration, HBT concentration, and enzyme concentration were the independent variable parameters that were optimized in this study.

The decolorization was calculated as (Equation (3)):

(3) $$Decolorization\;yield\;\left(\%\right)=\frac{Absorbance\;t_{0}-Absorbance\;t_{f}}{Absorbance\;t_{0}}\times 100$$

where “*Absorbance t*_0_” is the absorbance of the reaction mixture at the maximum wavelength (610 nm) of the dye before incubation with the enzyme and “*Absorbance t_f_*” is the absorbance (at the same wavelength) of the reaction mixture after incubation 1.5 mL reaction mixture in 100 mM tartrate buffer. The absorbance was measured by using a UV-Vis spectrophotometer (OPTIZEN, SMAC^gig^, Bangalore, India).

The decolorization rate was determined as follows (Equation (4)):

(4) $$Decolorization\;rate\;\left(\%\;/\min\right)=\frac{\;Decolorization\;yield\;(\%)}{incubation\;time\;(\min)}$$

### 4.6. Box-Behnken Design

As mentioned before, the Sirius grey decolorization yield in percentage (designated y_1_) and its decolorization rate in percentage of color removal per minute (designated y_2_) were considered as the experimentally studied responses (Equations (3) and (4), respectively).

The aim of this study was to determine the diverse influences of the four reaction studied factors, namely: HBT concentration (mM) (factor A), pH (factor B), initial dye concentration (mg/L) (factor C), and initial enzyme concentration (U/mL) (factor D), on the studied responses: decolorization yield (%) and rate (%/min). Table 5 presents the values of coded and uncoded levels used in this work.

**Table 5:** Coded and uncoded levels of tested factors.

	Coded Levels	−1	0	+1
Factor				
A: Initial HBT concentration (mM)		0.2	0.6	1
B: pH		3	4.5	6
C: Initial dye concentration (mg/L)		50	100	150
D: Initial enzyme concentration (U/mL)		0.2	0.6	1

Since each response can be influenced by one or more factors, the multivariate study using the Response Surface Methodology (RSM) with Box-Behnken design can be beneficial. In fact, the use of this methodology provides the possibility of determining the best polynomial multivariable model involving the coefficients calculation and statistical tests on one hand and the accurate identification of the optimum responses and the carrying out of the relative conditions on the other hand. 

Twenty-seven tested runs, repeated in triplicates, were analyzed via the Box–Behnken design and presented in Table 6 experimental response values as a function of experimental conditions.

The adopted model, in this case, had the following form with four studied factors (Equation (5)):

(5) $${\hat{y}}_{k}=\beta_{0}+\sum_{i=1}^{n}\beta_{i}\cdot x_{i_{k}}+\sum_{i=1}^{n}\beta_{ii}\cdot x_{i_{k}}^{2}+\sum_{i=1}^{n}\sum_{j>1}^{n}\beta_{ij}\cdot x_{i_{k}}\cdot x_{j_{k}}$$

where ${\hat{y}}_{k}$ are the modeled studied responses: decolorization yield of Sirius grey after 4 h in % (k = 1), and decolorization rate in % of color removal/min (k = 2); $\beta_{0}$, $\beta_{i}$, $\beta_{ii}$ and $\beta_{ij}$ are the model’s intercepts, linear, quadratic and interactions coefficients, respectively; x_i_ is the coded level of variable factors, n is the number of factors (n = 4).

**Table 6:** Box-Behnken’s design of experiment applied to the decolorization process of Sirius grey after 4 h of enzyme incubation and obtained responses.

Run	A:[HBT] *	B:pH *	C:[Dye] *	D:[Enzyme] *	y_1_: Yield (%)	y_2_: Rate (%/min)
1	−1	−1	0	0	49.91 ± 2.73	2.93 ± 0.15
2	1	−1	0	0	53.23 ± 1.41	2.87 ± 0.16
3	−1	1	0	0	56.07 ± 0.53	1.81 ± 0.02
4	1	1	0	0	72.14 ± 0.09	2.09 ± 0.01
5	0	0	−1	−1	77.63 ± 0.90	1.81 ± 0.08
6	0	0	1	−1	60.25 ± 0.88	1.29 ± 0.06
7	0	0	−1	1	85.04 ± 1.28	2.90 ± 0.24
8	0	0	1	1	76.74 ± 1.39	2.67 ± 0.02
9	−1	0	0	−1	49.71 ± 1.63	1.43 ± 0.20
10	1	0	0	−1	77.67 ± 0.04	1.68 ± 0.06
11	−1	0	0	1	62.78 ± 0.77	2.63 ± 0.06
12	1	0	0	1	86.15 ± 0.14	2.96 ± 0.03
13	0	−1	−1	0	55.32 ± 0.93	2.68 ± 0.02
14	0	+1	−1	0	68.12 ± 2.81	2.13 ± 0.07
15	0	−1	+1	0	51.41 ± 1.03	2.82 ± 0.09
16	0	+1	+1	0	50.97 ± 0.52	2.03 ± 0.03
17	−1	0	−1	0	73.49 ± 1.05	2.38 ± 0.13
18	1	0	−1	0	87.02 ± 1.33	2.51 ± 0.16
19	−1	0	+1	0	58.07 ± 0.80	1.83 ± 0.12
20	1	0	+1	0	79.86 ± 1.53	2.21 ± 0.12
21	0	−1	0	−1	49.51 ± 1.01	2.82 ± 0.07
22	0	+1	0	−1	40.14 ± 2.03	1.37 ± 0.22
23	0	−1	0	+1	64.32 ± 1.21	3.63 ± 0.12
24	0	+1	0	+1	65.57 ± 1.33	2.74 ± 0.02
25	0	0	0	0	79.69 ± 1.01	2.17 ± 0.22
26	0	0	0	0	79.01 ± 0.51	1.98 ± 0.08
27	0	0	0	0	79.19 ± 0.83	2.04 ± 0.11

* Factor levels are presented in coded values.

### 4.7. Design of Experiments and Statistical Analysis

The experimental Design, the model’s coefficients determinations, statistical analysis of the model quality and of the different factors’ influences, figures drawings, and the optimization protocol were performed within Minitab^®^ 19.2020.1 Statistical Software (64-bit) (© 2020 Minitab, LLC All rights reserved. Minitab^®^, the Minitab^®^ logo and CART^®^ are registered trademarks of Minitab, LLC in the United States and other countries). The models’ coefficients were determined using the least-squares method. Analysis of variance (ANOVA) was used to identify the level of significance of the studied model and the tested factors and their interactions with a confidence level of 95% (*p* < 0.05). The coefficient of determination (R^2^), the adjusted coefficient of determination (R^2^_adj_), and root mean square error (RMSE) were chosen to quantify the model fitting quality.

### 4.8. Phytotoxicity Assay

A phytotoxicity assay of the treated and untreated dye solutions was performed using radish seeds (*Raphanus sativus*). A Whatman filter paper was initially soaked with 2 mL of sterile distilled water, and then 5 mL of treated and untreated dye solutions were poured. Ten seeds were distributed on the paper, and the Petri dishes were incubated in the dark at 22 °C for 7 days. The germination index (GI) was calculated according to Equation (6):

(6) $$GI\left(\%\right)=\frac{\left(number\;of\;germinated\;seeds\;in\;treated\;seedlings)\times (length\;of\;roots\;of\;treated\;seedlings\right)}{\left(number\;of\;germinated\;seeds\;in\;control)\times (length\;of\;roots\;of\;control\;seedlings\right)}\times 100$$

## 5. Conclusions

Basidiomycetes secrete a range of oxidases, including laccases and peroxidases, that could transform a number of pollutants, including recalcitrant dyes, into non-toxic molecules. Because of the high redox potential of dye molecules or their incapacity to reach active sites, small redox mediators are often required for optimal decolorization. In addition, several other reaction parameters, including pH, dye concentration, and enzyme concentration, also have to be optimized. In this study, it was shown that response surface methodology was an effective method to reach a high decolorization rate of the recalcitrant four azo bonds dye: Sirius grey. This study confirmed that the laccase-HBT system could be a promising tool for the decolorization and detoxification of textile dyes.

## Figures and Tables

**Table 1:** ANOVA results of Sirius grey decolorization yield.

Source	DF	SS	MS	F	*p*
Regression	14	13290.4	949.3	51.63	<0.001 ***
Linear	4	6062.2	1515.5	82.43	<0.001 ***
A: Initial [HBT]	1	2810.8	2810.8	152.87	<0.001 ***
B: pH	1	214.7	214.7	11.68	0.001 ***
C: Initial [Dye]	1	1200.9	1200.9	65.31	<0.001 ***
D: Initial [Enzyme]	1	1835.8	1835.8	99.85	<0.001 ***
Square	4	6761.1	1690.3	91.93	<0.001 ***
A × A	1	197.8	197.8	10.76	0.002 **
B × B	1	6202.5	6202.5	337.34	<0.001 ***
C × C	1	26.5	26.5	1.44	0.234
D × D	1	374.7	374.66	20.38	<0.001 ***
Interaction	6	467.1	77.86	4.23	0.001 ***
A × B	1	121.9	121.92	6.63	0.012 *
A × C	1	51.3	51.25	2.79	0.100
A × D	1	15.8	15.80	0.86	0.357
B × C	1	131.6	131.61	7.16	0.009 **
B × D	1	84.6	84.64	4.60	0.036 *
C × D	1	61.9	61.93	3.37	0.071
Error	66	1213.5	18.39		
Total	80	14503.9			

DF: degree of freedom; SS: sum of squares; MS: mean square; F: Fisher value; *p*: probability value; *** Very highly significant (*p* < 0.001); ** Very significant (*p* < 0.010); * Significant (*p* < 0.050).

**Table 2:** ANOVA results of Sirius grey decolorization rate.

Source	DF	SS	MS	F	*p*
Regression	14	24.348	1.739	57.96	<0.001 ***
Linear	4	21.498	5.375	179.11	<0.001 ***
A: Initial [HBT]	1	0.420	0.420	13.99	<0.001 ***
B: pH	1	7.767	7.767	258.85	<0.001 ***
C: Initial [Dye]	1	0.612	0.612	20.41	<0.001 ***
D: Initial [Enzyme]	1	12.699	12.699	423.19	<0.001 ***
Square	4	2.369	0.592	19.73	<0.001 ***
A × A	1	0.026	0.026	0.86	0.357
B × B	1	2.137	2.137	71.22	<0.001 ***
C × C	1	0.015	0.015	0.51	0.479
D × D	1	0.214	0.214	7.14	0.009 **
Interaction	6	0.481	0.080	2.67	0.022 *
A × B	1	0.086	0.086	2.85	0.096
A × C	1	0.048	0.048	1.61	0.209
A × D	1	0.005	0.005	0.18	0.672
B × C	1	0.041	0.041	1.36	0.248
B × D	1	0.236	0.236	7.88	0.007 **
C × D	1	0.065	0.065	2.15	0.147
Error	66	1.981	0.030		
Total	80	26.329			

DF: degree of freedom; SS: sum of squares; MS: mean square; F: Fisher value; *p*: probability value; *** Very highly significant (*p* < 0.001); ** Very significant (*p* < 0.010); * Significant (*p* < 0.050).

**Table 3:** Properties of the azo dye Sirius grey GB (Direct Black 76), DMP, and HBT.

Properties	Sirius Grey GB	DMP	HBT
CAS number	6409-87-6	91-10-1	12333-53-9
Molecular weight (g mol^−1^)	1193.99	154.16	135.12
EC Number	241-164-5	202-041-1	219-989-7
CI	35,865	-	-
Empirical formula	C_46_H_27_N_11_O_15_S_4_.4Na	(CH_3_O)_2_C_6_H_3_OH	C_6_H_5_N_3_O · xH_2_O
Other names	Direct black 76	Pyrogallol 1,3-dimethyl ether	HOBt Hydrate
λ_max_ (nm)	610	-	-
Number azo bonds	4	-	-
Purity (%)		99	97

**Table 4:** GenBank sequences are used to calculate the phylogenetic tree.

Species	Culture Collection Designation	ITS Accession Number
*C. gallica*	BS9	OR234862
*C. gallica*	CBS547.50	MH856754
*C. gallica*	CBS429.34	MH855593
*C. gallica*	CBS428.34	MH855592
*C. trogii*	LE-BIN_3828	OQ053212
*C. trogii*	KM096	OQ450434
*C. trogii*	Han474	ON796506
*Funalia subgallica*	Dai6329	KC867386
*F. subgallica*	Cui6317	KC867384
*F. subgallica*	BJFC004185	NR_174714

## Data Availability

The authors declare data transparency.
